# Pediatric trauma and emergency surgery: an international cross-sectional survey among *WSES* members

**DOI:** 10.1186/s13017-022-00473-5

**Published:** 2023-01-13

**Authors:** Martin Reichert, Massimo Sartelli, Ingolf H. Askevold, Jaqueline Braun, Markus A. Weigand, Matthias Hecker, Vanni Agnoletti, Federico Coccolini, Fausto Catena, Winfried Padberg, Jens G. Riedel, Andreas Hecker, The WSES pediatric emergency surgery collaboration group, Agron Dogjani, Akira Kuriyama, Alberto Porcu, Aleix Martínez-Pérez, Alessandro Coppola, Alessandro Spolini, Alessio Giordano, Alexandros Kyriakidis, Ali Yasen Y. Mohamedahmed, Anastasia Vasilopoulou, Andee Dzulkarnaen Zakaria, Andrea Balla, Andreas Fette, Andrey Litvin, Anna Guariniello, Arda Isik, Aristotelis Kechagias, Ashrarur Rahman Mitul, Belinda De Simone, Biagio Zampogna, Bruno Sensi, Carlo Gazia, Charalampos Seretis, Cristine Brooke, Davide Luppi, Diego Coletta, Diego Sasia, Diletta Corallino, Dimitrios Chatzipetris, Dimitrios Schizas, Eftychios Lostoridis, Elmuiz A. Hsabo, Emmanouil Kaouras, Emmanuel Schneck, Enrico Pinotti, Evgeni Dimitrov, Fabrizio D’Acapito, Federica Saraceno, Fikri Abu-Zidan, Francesca Maria Silvestri, Francesco Favi, Francesco Fleres, Francesk Mulita, Gabriela Nita, Gennaro Martines, Gennaro Mazzarella, Gennaro Perrone, Giorgio Giraudo, Giulia Bacchiocchi, Giulio Argenio, Giuseppe Brisinda, Giuseppe Currò, Giuseppe Palomba, Gustavo P. Fraga, Hytham K. S. Hamid, Ioannis Katsaros, Ionut Negoi, Joel Noutakdie Tochie, Justin Davies, Kenneth Y. Y. Kok, Konstantinos G. Apostolou, Konstantinos Lasithiotakis, Konstantinos Tsekouras, Larysa Sydorchuk, Leandro Siragusa, Leonardo Solaini, Luca Ferrario, Luis Buonomo, Maciej Walędziak, Mahir Gachabayov, Maloni Bulanauca, Manish Kumar Agrawal, Marco Ceresoli, Maria Chiara Ranucci, Maria Petridou, Mario D’Oria, Massimiliano Veroux, Maximos Frountzas, Michel Paul Johan Teuben, Miklosh Bala, Mirja Amadea Minger, Miroslava Gonçalves, Natasha Sharma, Nicolò Tamini, Noushif Medappil, Orestis Ioannidis, Pietro Bisagni, Razrim Rahim, Ricardo Alessandro Teixeira Gonsaga, Roberta Ragozzino, Roberto Bini, Roberto Cammarata, Ruslan Sydorchuk, Salomone Di Saverio, Selmy S. Awad, Semra Demirli Atici, Serhat Meric, Sharfuddin Chowdhury, Sofia Xenaki, Tadeja Pintar, Teresa Perra, Timothy C. Hardcastle, Valerio Voglino, Varut Lohsiriwat, Victor Kong, Voskidis Christos, Wietse Zuidema

**Affiliations:** 1 Department of General, Visceral, Thoracic, Transplant and Pediatric Surgery, University Hospital of Giessen, Rudolf-Buchheim-Strasse 7, 35392 Giessen, Germany; 2 Department of Surgery, Macerata Hospital, Macerata, Italy; 3 Department of Anesthesiology, Heidelberg University Hospital, Heidelberg, Germany; 4 Department of Pulmonary and Critical Care Medicine, University Hospital of Giessen and Marburg Lung Center (UGMLC), University Hospital of Giessen, Giessen, Germany; 5 Anesthesia and Intensive Care Unit, Maurizio Bufalini Hospital, Cesna, Italy; 6 Department of General, Emergency and Trauma Surgery, Pisa University Hospital, Pisa, Italy; 7 Department of Emergency Surgery, Parma Maggiore Hospital, Parma, Italy; 8 Division of Pediatric Surgery, Department of General, Visceral, Thoracic, Transplant and Pediatric Surgery, University Hospital of Giessen, Giessen, Germany

**Keywords:** Emergency surgery, Trauma surgery, Emergency, Appendicitis, WSES, Pediatric surgery, Children, Injury, Pediatric trauma

## Abstract

**Background:**

In contrast to adults, the situation for pediatric trauma care from an international point of view and the global management of severely injured children remain rather unclear. The current study investigates structural management of pediatric trauma in centers of different trauma levels as well as experiences with pediatric trauma management around the world.

**Methods:**

A web-survey had been distributed to the global mailing list of the *World Society of Emergency Surgery* from 10/2021–03/2022, investigating characteristics of respondents and affiliated hospitals, case-load of pediatric trauma patients, capacities and infrastructure for critical care in children, trauma team composition, clinical work-up and individual experiences with pediatric trauma management in response to patients´ age. The collaboration group was subdivided regarding sizes of affiliated hospitals to allow comparisons concerning hospital volumes. Comparable results were conducted to statistical analysis.

**Results:**

A total of 133 participants from 34 countries, i.e. 5 continents responded to the survey. They were most commonly affiliated with larger hospitals (> 500 beds in 72.9%) and with level I or II trauma centers (82.0%), respectively. 74.4% of hospitals offer unrestricted pediatric medical care, but only 63.2% and 42.9% of the participants had sufficient experiences with trauma care in children ≤ 10 and ≤ 5 years of age (*p* = 0.0014). This situation is aggravated in participants from smaller hospitals (*p* < 0.01). With regard to hospital size (≤ 500 versus > 500 in-hospital beds), larger hospitals were more likely affiliated with advanced trauma centers, more elaborated pediatric intensive care infrastructure (*p* < 0.0001), treated children at all ages more frequently (*p* = 0.0938) and have higher case-loads of severely injured children < 12 years of age (*p* = 0.0009). Therefore, the majority of larger hospitals reserve either pediatric surgery departments or board-certified pediatric surgeons (*p* < 0.0001) and in-hospital trauma management is conducted more multi-disciplinarily. However, the majority of respondents does not feel prepared for treatment of severe pediatric trauma and call for special educational and practical training courses (overall: 80.2% and 64.3%, respectively).

**Conclusions:**

Multi-professional management of pediatric trauma and individual experiences with severely injured children depend on volumes, level of trauma centers and infrastructure of the hospital. However, respondents from hospitals at all levels of trauma care complain about an alarming lack of knowledge on pediatric trauma management.

**Supplementary information:**

The online version contains supplementary material available at 10.1186/s13017-022-00473-5.

## Background

Pre-clinical classification, in-hospital survey, management and treatment of trauma patients is basically a well-organized and choreographed continuum, whose principles are fixed in evidence-based national guidelines [1, 2], recommendations from diverse medical societies [3–18, www.aast.org], as well as educational programs [19]. Driven by current evidences, various recommendations give important implications on the classification and emergency management of severely injured trauma patients. Furthermore, they provide a concrete evidence-based guide for either non-operative, interventional or operative treatment options for multiple and complex injuries [1–19, www.aast.org]. Management and therapeutic decision-making in diverse trauma situations had been well described with high levels of evidence and are clinical routine in emergency departments around the world—for adult patients. In contrast, the incidence of severe trauma in children is certainly lower [2, 19–21], but, “injury remains the most common cause of death and disability in childhood” and “injury morbidity and mortality […] making trauma the most serious public health and health care problem in this population” [19]. These two introducing statements from the *Advanced Trauma Life Support ®* (ATLS ®) manual (tenth edition) [19] underline the importance of adequate reaction and therapeutic decisions in pediatric trauma, which vice versa improves short-term mortality and long-term outcome [22–24]. However, children are not small adults [2, 19, 25]. Trauma classification systems, diagnostic and therapeutic options as well as surgical techniques or intra-operative considerations in the emergency trauma setting majorly differ between children and adults [2, 6, 9, 19, 26–28]. Despite adequate pre-hospital patient selection and consecutive allocation of severely injured pediatric patients to more experienced trauma centers by rescue services [2, 29], members from trauma centers at any level should be aware of treatment principles and early resuscitation for special cases of pediatric trauma. Knowledge on the principles of pediatric trauma care or inclusion of specialized pediatric surgeons into multi-professional treatment considerations of severely injured children consecutively leads not only to decreased mortality but also to decreased rates of (unnecessary) surgery, for example reduced rates of splenectomies and higher rates of organ-preserving therapy [2, 23, 24, 29–33]. Thereby, recommendations from adult trauma guidelines cannot simply applied to pediatric trauma situations. However, pediatric trauma is insufficiently covered by general trauma guidelines and consecutively high evidence-based recommendations for pediatric trauma management are widely lacking [1, 2, 6, 19]. Furthermore, studies including especially pediatric patients are also rare on sustaining evidence-based trauma protocols in relation to outcome. In consequence, the situation of pediatric trauma care from an international point of view remains rather unclear. Situations of severe pediatric trauma induce stress, have psychological and emotional effects on trauma team members. The current study investigates structural management of pediatric trauma in hospitals of different volumes and consecutively centers of different trauma levels, experiences with and necessity of educational programs for pediatric trauma management. The responses are set in relation to sizes of hospitals and ages of the severely injured children. This follows the aim to enhance preparedness for treatment of severely injured children, to reduce concerns and fears against pediatric trauma and to improve quality of trauma care in children, patient morbidity and mortality. By this survey, the *World Society of Emergency Surgery* (WSES) claims to gain global information on pediatric trauma care. The results could lead to the initiation of educational programs for pediatric trauma management and to underline the necessity for a WSES-driven guideline program.

## Methods

As described previously [34, 35], an online survey was designed by a core group of investigators of the study group. Google Forms (Google LLC, Mountain View, California, USA) was used as the platform for the survey. After the survey was approved by the WSES project steering committee, this cross-sectional survey study was distributed in October 2021 to the global mailing list of WSES members. The survey was closed in March 2022.

The survey was designed, the survey study was conducted, and the results were analyzed and reported following the *CHERRIES* statement [36].

The survey consists of single-choice items as well as one open-answer question. The items of the present questionnaire are organized in five sections: (1) recording the characteristics of respondents and their affiliated hospitals, (2) volume of pediatric trauma patients, (3) individual capacities and infrastructure for pediatric trauma and surgical emergency care in children, (4) in-hospital trauma team composition, clinical work-up and team training for pediatric trauma and pediatric surgical emergency care and (5) individual experiences with pediatric trauma care and emergency surgery in children in response to patients´ age.

After closing the survey, the results were checked for duplicates. Results of the total study cohort are presented descriptively within the Figures of the manuscript.

Results of items, that were comparable, were conducted to statistical analyses. Furthermore, the collaboration group was subdivided with regard to sizes of affiliated hospitals. ≤ 500 in-hospital beds indicated the smaller hospitals, > 500 in-hospital beds indicated the larger hospitals. Comparisons of survey results allow to interpret the current situation in pediatric traumatology and emergency surgery concerning individual hospital volumes. Results of subgroup analyses are presented in Tables.

Statistical analyses were performed using GraphPad Prism (Version 9 for Windows, GraphPad Software, San Diego, CA, USA, www.graphpad.com). For descriptive statistics, categorical data were analyzed using Fishers exact test or Pearson’s X^2^ test. p values ≤ 0.05 were considered to indicate statistical significance. Because of the exploratory character of the study, no adjustments of p values were performed. Data are given as n respondents and / or percentages of the participants.

## Results

### Characteristics of participants and their affiliated hospitals

**Fig. 1:**
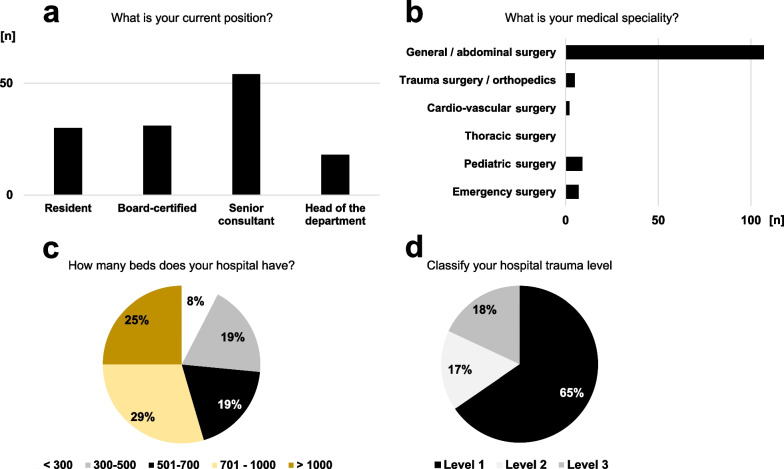
Characteristics of the participants and their affiliated hospitals. The current position (**a**) and the medical profession (**b**) of the respondents. The volume of the affiliated hospitals (**c**) and the level of the trauma centers from I to III (**d**). The level of the trauma centers was defined in accordance with the current definition from the American College of Surgeons and American Trauma Society [www.amtrauma.org/page/TraumaLevels, accessed May 2nd, 2022 and 37]

A total of 133 health care specialists in emergency medicine and emergency surgical patient care from several 34 countries (5 continents, approximately two-thirds from Europe and 21% from Asia) around the world completed the survey (Additional file 1). Most of the participants work in larger hospitals with more than 500 beds (72.9%). They were affiliated with level one or level two trauma centers (82.0%), defined by the American College of Surgeons (ACS) and the American Trauma Society (ATS) as a most experienced trauma team in the emergency room, which is constantly available and able to initiate treatment of every aspect of injury [www.amtrauma.org/page/TraumaLevels, accessed May 2nd, 2022 and 37]. The vast majority of the respondents came from general and abdominal surgery (80.5%) and were well-experienced personnel, including at least board-certified surgeons in 77.4% (Fig. 1).

### Individual infrastructure for pediatric trauma and surgical emergency care

**Fig. 2:**
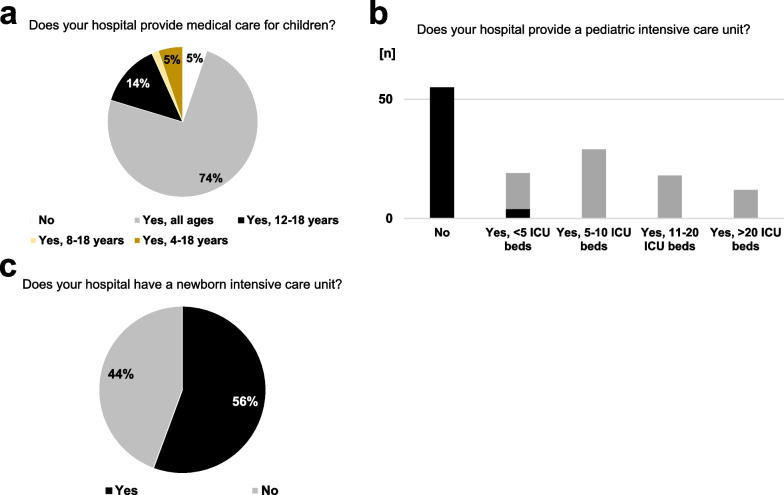
Infrastructure for the care of critically-ill pediatric patients. Most of the affiliated hospitals provide medical care for children (**a**), but approximately half of the hospitals lack a pediatric (**b**) or newborn intensive care unit (**c**). Figure 2b: gray bars indicate hospitals, which also treat newborns; black bars indicate hospitals, which do not treat newborns

**Fig. 3:**
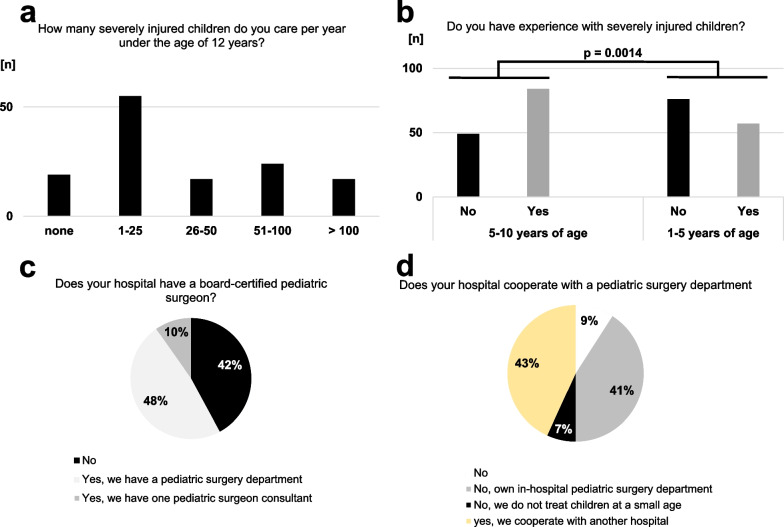
Individual and institutional experiences with pediatric trauma care of participants and their affiliated hospitals. The trauma center volume with regard to the treatment of severely injured children under 12 years of age (**a**). Significantly less participants were experienced with treatment of severely injured children below the age of 5 years (**b**, *p* = 0.0014 versus 5–10 years of age). In-hospital availability of specialized pediatric surgeons (**c**) or a department of pediatric surgery (**d**)

Medical care for children at any age was provided by 74.4% of the affiliated hospitals. However, pediatric patient care was not offered in 5.3% of the affiliated hospitals. In most other cases, the ability to treat pediatric patients was stratified by patient age. In this line, 41.4% or 44.4% of the affiliated hospitals were not equipped with general pediatric intensive care units (ICU) or units for intensive care of newborns, respectively. Vice versa, if a pediatric ICU was available, intensive care of newborns was also possible in most cases. Nevertheless, high-volume pediatric ICUs with more than 10 beds had rarely been reported in the affiliated hospitals of the participants of the survey (Fig. 2). In this line, the vast majority reported a rather low case load (*n = *1–25 per year) of severely injured children below the age of 12 years. Experiences of the study group with severely injured children declined dramatically by patient age: while 63.2% of the participants had any experiences with surgical trauma care in children with an age of 5–10 years, this rate decreased to 42.9% in children below the age of 5 (*p* = 0.0014). Facing this situation, some 57.9% or 42.9% of the participants work together with specialized pediatric surgeons either from their affiliated hospital or by cooperation with pediatric surgeons from other hospitals in cases of severely injured children, respectively (Fig. 3).

### Team building and team training for pediatric surgical emergency care

**Fig. 4:**
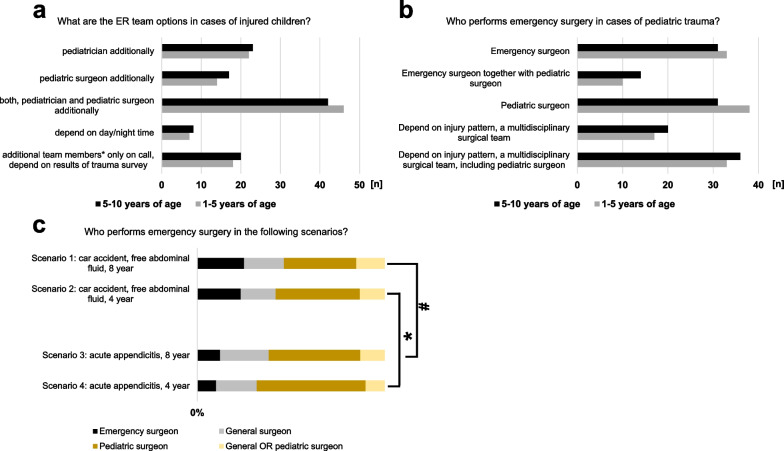
Team considerations in pediatric emergency situations. Emergency room (ER) and surgical team compositions in cases of injured children (**a** and **b**, respectively). Scenarios: trauma surgery versus appendicitis in children at different ages (**c**). * indicates *p* = 0.0210. # indicates *p* = 0.0485

The structure of in-hospital trauma teams in their affiliated hospitals varied strongly between the respondents. One-third of the participants reported that both pediatricians and pediatric surgeons are the regular team members during the in-hospital trauma survey for severely injured children regardless of patient age. Otherwise, adjustments of the standard trauma team were possible upon results of the primary trauma survey and in relation to day or night time (Fig. 4).

Similar differences in initial trauma teams were recorded for the consecutive question "Who performs emergency surgery for pediatric trauma in patients younger than 10 or 5 years of age?". While approximately one fourth of the respondents stated that emergency surgery in pediatric trauma patients is routinely performed either by emergency surgeons or by specialized pediatric surgeons, in half of the affiliated hospitals complex severely injured children undergo surgery by a multidisciplinary team, which includes specialized pediatric surgeons (Fig. 4). In the opposite to surgical care of younger patients with acute appendicitis, the patients´ age has no strong statistic effect on team considerations in emergency surgery for trauma.

**Fig. 5:**
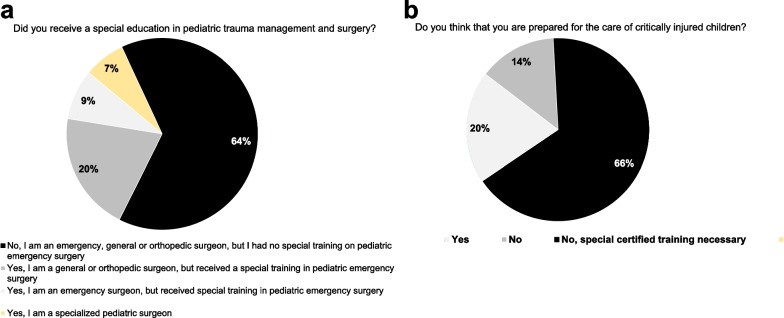
Preparedness for care of severely injured pediatric patients. The majority of emergency surgeons worldwide received neither a special education in pediatric trauma management or surgery (**a**) nor feel sufficiently prepared for the management and surgical treatment of critically injured pediatric trauma patients (**b**)

**Fig. 6:**
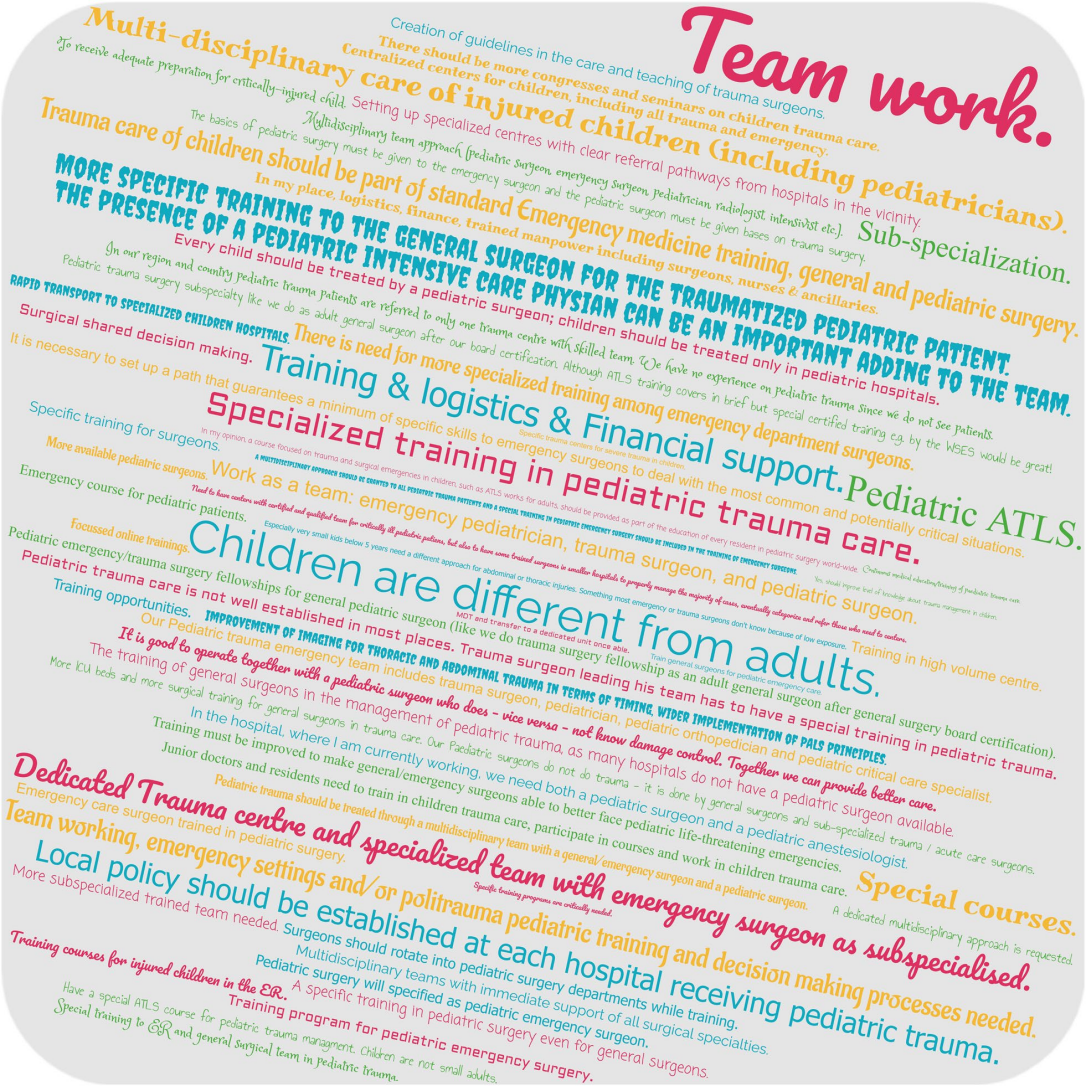
What might enhance preparedness of trauma teams for severely injured children and quality of pediatric trauma care in the future? Answers from the respondents collected in a word cloud

Last and most relevant, the majority of participants of the present survey (80.2%) do not feel sufficiently prepared for the (surgical) treatment of severely injured children and 64.3% stated the urgent need for special training in pediatric trauma care and emergency surgery in children (Fig. 5). The word cloud in Fig. 6 collects the answers on the question, what might enhance preparedness of trauma teams for severely injured children and consequently quality of pediatric trauma care in the future.

### Hospital size effects on pediatric emergency and trauma care

The hospital size was stratified by numbers of in-hospital beds. A bed count of more than 500 was decided to separate larger from smaller hospitals. 36 (27.1%) participants work in smaller hospitals (≤ 500 in-hospital beds), vice versa, 97 (72.9%) respondents work in larger hospitals (> 500 in-hospital beds). Larger hospitals were more likely being affiliated with more advanced trauma centers including full trauma care being continuously available with well-experienced staff (level 1: 33.3% versus 77.3%, *p* < 0.0001) as well as with more elaborated pediatric intensive care infrastructure (*p* < 0.0001). Although not significant, treatment of children of all ages was more frequently feasible in larger hospitals by tendency (58.3% versus 80.4%, *p* = 0.0938); the estimated case load of more severely injured children below the age of 12 was considerably higher in larger hospitals (*p* = 0.0009). Therefore, the majority of larger hospitals reserve either an independent department for pediatric surgery or at least one board-certified pediatric surgeon consultant (22.2% versus 71.1%, *p* < 0.0001, Table 1). Nevertheless, a significant proportion of the respondents, who are themselves specialists in surgical emergency and trauma care, stated that they have no sufficient experiences with severely injured children. This situation is dramatically aggravated by lower patient age and is more evident in participants from smaller hospitals (Table 1 and 2). Interestingly, in-hospital trauma teams as well as surgical teams involved in the treatment of pediatric trauma are composed primarily more multidisciplinary including pediatricians and specialized pediatric surgeons in larger hospitals, whereas a specific age effect of injured children (1–5 years versus 5–10 years of age) was not observed herein (Tables 3 and 4).

**Table 1:** Spectrum and expertise with regard to the size and volume of affiliated hospitals

Items (question & answer)	≤ 500 in-hospital beds *n = *36 (27.1%)		> 500 in-hospital beds *n* = 97 (72.9%)		*p *value
	(n)	n.a	(n)	n.a	
**Trauma center**^**#**^		0		0	*p* < 0.0001
Level 1	12		75		
Level 2	13		9		
Level 3	11		13		
**Does your hospital regularly provide medical care for children?**		0		0	*p* = 0.0938
No	2		5		
Yes, all ages (0–18 years)	21		78		
Yes, at an age of 4–18 years	3		4		
Yes, at an age of 8–18 years	1		1		
Yes, at an age of 12–18 years	9		9		
**Does your hospital provide a pediatric ICU?**		0		0	*p* < 0.0001
No	26		29		
Yes, < 5 ICU beds	8		11		
Yes, 5–10 ICU beds	1		28		
Yes, 11–20 ICU beds	1		17		
Yes, > 20 ICU beds	0		12		
**How many severely injured children do you care per year at an age < 12 years?**		0		1	*p* = 0.0009
None	7		12		
1–25	24		31		
26–50	1		16		
51–100	2		22		
> 100	2		15		
**Does your hospital have a board-certified pediatric surgeon?**		0		0	*p* < 0.0001
No	28		28		
Yes, own department	3		61		
Yes, one pediatric surgeon consultant	5		8		
**Do you have experiences with injured children?**		0		0	
**5–10 years of age**					*p* = 0.0086
No	20 *		29*		
Yes	16		68		
**1–5 years of age**					*p* = 0.0002
No	30*		46*		
Yes	6		51		

#Regarding the American College of Surgeons and American Trauma Society. n.a. = no answer. ICU = Intensive care unit. *Significant difference (p ≤ 0.05) by patient age (1–5 years versus 5–10 years of age)

**Table 2:** Preparedness for pediatric trauma of the collaborators with regard to the size and volume of affiliated hospitals

Items (question & answer)	≤ 500 in-hospital beds *n = *36 (27.1%)		> 500 in-hospital beds *n = *97 (72.9%)		*p *value
	(*n*)	n.a	(*n*)	n.a	
**Did you receive a special training in pediatric emergency management and surgery?**		1		3	*p* = 0.0075
No	29		54		
Yes	6		40		
**Do you feel prepared for management, surgery and treatment of an severely injured child?**		1		1	*p* = 0.0024
No	34		71		
Yes	1		25		

n.a. = no answer

**Table 3:** Trauma team considerations with regard to the size and volume of the affiliated hospitals

Items (question & answer)	≤ 500 in-hospital beds *n = *36 (27.1%)		> 500 in-hospital beds *n = *97 (72.9%)		*p *value
	(*n*)	n.a	(*n*)	n.a	
**Trauma team in cases of injured children?**					
**5–10 years of age**		0		2	*p* = 0.0043
Routine team	5^ø^		16^ø^		
Pediatrician additionally	12		11		
Pediatric surgeon additionally	4		13		
Pediatrician AND pediatric surgeon additionally	6		36		
Depend on day/night time	0		8		
Additional team members on-call, depend on results of trauma survey	9		11		
**1–5 years of age**		1		1	*p* = 0.0147
Routine team	5^ø^		19^ø^		
Pediatrician additionally	10		12		
Pediatric surgeon additionally	3		11		
Pediatrician AND pediatric surgeon additionally	8		38		
Depend on day/night time	0		7		
Additional team members on-call, depend on results of trauma survey	9		9		
**Who performs emergency surgery in cases of injured children?**					
**5–10 years of age**		0		1	*p* < 0.0001
Emergency surgeon	18^ø^		13^ø^		
Emergency surgeon together with the pediatric surgeon	4		10		
Pediatric surgeon	6		25		
Depend on injury, multidisciplinary surgical team	6		14		
Depend on injury, multidisciplinary surgical team including pediatric surgeons	2		34		
**1–5 years of age**		1		1	*p* = 0.0053
Emergency surgeon	16^ø^		17^ø^		
Emergency surgeon together with the pediatric surgeon	2		8		
Pediatric surgeon	8		30		
Depend on injury, multidisciplinary surgical team	6		11		
Depend on injury, multidisciplinary surgical team including pediatric surgeons	3		30		

n.a. = no answer. ICU = Intensive care unit. Ø no difference (p > 0.05) by patient age (1–5 years versus 5–10 years of age)

**Table 4:** Surgical team considerations for pediatric surgical emergency scenarios with regard to the size and volume of affiliated hospitals

Items (Question & answer)	≤ 500 in-hospital beds *n = *36 (27.1%)		> 500 in-hospital beds *n = *97 (72.9%)		*p *value
	(*n*)	n.a	(*n*)	n.a	
**Who performs trauma surgery?**					
**Scenario 1: car accident, free abdominal fluid in a 8 year old child?**		1		0	*p* = 0.0004
Emergency surgeon	10^ø^		23^ø^		
General surgeon	15		13		
Pediatric surgeon	5		46		
General OR pediatric surgeon	5		15		
**Scenario 2: car accident, free abdominal fluid in a 4 year old child?**		3		1	*p* = 0.0002
Emergency surgeon	9^ø^		21^ø^		
General surgeon	14		10		
Pediatric surgeon	8		50		
General OR pediatric surgeon	2		15		
**Who performs emergency surgery?**					
**Scenario 3: acute appendicitis in a 8 year old child?**		5		5	*p* = 0.0025
Emergency surgeon	5^ø^		10^ø^		
General surgeon	15		17		
Pediatric surgeon	7		53		
General OR pediatric surgeon	4		12		
**Scenario 4: acute appendicitis in a 4 year old child?**		2		2	*p* = 0.0046
Emergency surgeon	5^ø^		8^ø^		
General surgeon	14		14		
Pediatric surgeon	13		62		
General OR pediatric surgeon	2		11		

n.a. = no answer. Ø no difference (p > 0.05) by patient age (1–5 years versus 5–10 years of age)

## Discussion

Basically, the present study dramatically confirms the expected conclusion: Everyone is involved in some way in the treatment of injured children, but the minority is really sufficiently prepared for pediatric trauma management. As expected, the sizes of trauma centers have an influence on the experience level of the emergency surgeon in pediatric trauma management: Although even 29.9% and 47.4% of the respondents from larger hospitals have no experiences with trauma management of children below the age of 10 years and below the age of 5 years, respectively, significantly more emergency surgeons from smaller hospitals were vice versa inexperienced with pediatric trauma management at all. Pediatric trauma management in larger hospitals is performed more multi-professionally according to the trauma level definition from the ACS and ATS [www.amtrauma.org/page/TraumaLevels, accessed May 2nd, 2022 and 37]. Participants from larger hospitals were more commonly trained in pediatric trauma management and, furthermore, pediatricians as well as specialized pediatric surgeons play a major role in the treatment of severely injured children in these larger, even more experienced centers.

Nevertheless, through the global perspective centralization of pediatric trauma care is difficult. The treatment of critically injured children is typically covered worldwide by both smaller and larger trauma centers. In many countries, a comprehensive trauma care for children with highly experienced and specialized pediatric surgeons is not well organized even for the early resuscitation phase of trauma care. This is particularly the critical situation in rural areas, where also hospitals without dedicated pediatric trauma services are confronted with these special pediatric trauma cases [37]. Our study reflects this problem, as also trauma teams from smaller hospitals with even inexperienced trauma centers are involved in the initial treatment or urgent resuscitation of pediatric trauma patients with nonsignificant numbers per year (1–25 severely injured children in 66.7% of these cases, see Table 1).

Although the response rate with 133 participants from the global mailing list of the international WSES was rather low and reflects a major limitation of our study, the results of the survey give important implications on the question: How experienced are emergency surgeons worldwide in pediatric trauma care. Though the vast majority of participants from smaller hospitals (97.1%) do not feel enough and sufficiently prepared for the treatment of severely injured children. Nevertheless, even 74.0% of the respondents from larger hospitals do not feel prepared adequately for appropriate management of severely injured children, but they can fall back and have access to sufficient back-up consisting of specialized pediatric surgeons, pediatricians and high-volume pediatric ICU in their affiliated trauma centers providing total care of every aspect of injury [www.amtrauma.org/page/TraumaLevels, accessed May 2nd, 2022 and 37].

Highly evidence-based and universal recommendations on pediatric trauma management from local or international medical societies analogous to adult trauma are widely lacking at present. To face this dilemma in pediatric emergency trauma care, the *Committee on Trauma* from the *American College of Surgeons* gives advices for defining pediatric trauma centers and pediatric trauma teams, which might facilitate referral considerations of the involved pre-hospital rescue service [22, 23, 37, 38]. Whether this closes the gap between the lack of evidence, the low experience especially in small hospitals and the clinical outcome of pediatric trauma patients remains to be determined in the future. However, our data show impressively that there are situations, when even smaller hospitals affiliated with lower level (adult) trauma centers without broad experience in emergency care of pediatric patients have to provide primary care of severely injured children, especially in areas where pediatric emergency and trauma services are scarce [37]. It is alarming that our study shows a dramatic lack of education, practical training as well as tactical and organizational deficiencies for these pediatric cases. Consequently, surgeons from smaller hospitals with less experienced trauma centers should also increase their knowledge of principles and treatment considerations in pediatric trauma management. Vice versa, this will not only reduce concerns and fears against situations with severely injured children, but also might improve outcomes of pediatric trauma patients. The worldwide recognized educational ALTS ® course has now implemented a specific section about pediatric trauma [19]. Guidelines, which are available for traumatic injuries in adults, should be revised and edited for pediatric trauma patients by respective national as well as international medical societies, like the *World Society for Emergency Surgeons* had done for various situations of severe trauma (e.g., spleen trauma, liver trauma, duodeno-pancreatic and biliary tree trauma or kidney and uro- trauma) [5, 6, 9–11, 39]. This might improve theoretical education and knowledge of trauma team members, especially trauma surgeons involved in pediatric trauma management. On a more local basis trans-regional level I or II trauma centers from larger hospitals should provide practical courses, consultations or fellowships for trauma team members and trauma surgeons from more unexperienced trauma centers or smaller hospitals to enhance and exercise their theoretical knowledge in “real life”. Both might increase and strengthen the preparedness for management of trauma and severe injuries in pediatric patients even of trauma teams, who are not regularly confronted with severely injured children.

Despite these important aspects in the care of pediatric trauma from the global perspective, our study has some other limitations that must be listed besides the low response rate mentioned above. Precise conclusions about differences between individual countries or specific regions cannot be drawn due to the low return rate. Furthermore, the responses are based on subjective opinions and feelings of the participants and, for example, the answers to the infrastructural questions were not officially verified. For these reasons, it was decided to use hospital size (≤ versus > 500 beds) rather than trauma center level regarding the ATS to stratify hospitals, even though the parameter is certainly somewhat less accurate in capturing the quality of care for trauma patients. Although the number (≤ versus > 500 beds) was chosen arbitrarily, it can be anticipated and it is shown by results presented here that rather the larger hospitals are associated with more advanced trauma centers. Finally, the lack of external validation of the survey should certainly be seen as a minor limitation of the work. To take this into account, the survey was designed and internally validated by core investigators of this work and then, revised and validated for the international professional society members by the WSES steering committee representatives.

## Conclusions

In conclusion, our study shows that emergency surgeons from both high and low level trauma centers report an alarming lack of knowledge on pediatric trauma care. Multi-professional management of pediatric trauma patients and the individual experience with severely injured children is strongly dependent on the volume, the level of the trauma center and infrastructure of the specific hospital. However, smaller hospitals with less experienced trauma centers are also confronted with pediatric trauma patients. This underlines the importance of theoretical education and practical training programs for appropriate management of pediatric trauma and primary survey or resuscitation as well as early induction of adequate therapy of severely injured children. Recommendation, guidance of appropriate therapy, teaching and training might be the future mission of national and international medical societies as well as trans-regional well-experienced pediatric trauma centers in the perspective of global improvements in pediatric trauma care.

## Supplementary information

**Additional file 1.** The WSES pediatric emergency surgery collaboration group (only those who agree are listed as collaborators).

## Data Availability

The datasets used and/or analyzed during the current study are available from the corresponding author on reasonable request.

## Abbreviations

ACS: American College of Surgeons
ATS: American Trauma Society
CHERRIES: The checklist for reporting results of Internet E-Surveys
ICU: Intensive care unit
WSES: World Society of Emergency Surgery

## References

[1] Bouillon B. S3 – Leitlinie Polytrauma/Schwerverletztenbehandlung, AWMF Register-Nr. 012/019. 2016;

[2] Schmittenbecher PP. S2k - Leitlinie Polytraumaversorgung im Kindesalter, AWMF Register-Nr. 006–120. 2020;

[3] Kozar RA, Crandall M, Shanmuganathan K, Zarzaur BL, Coburn M, Cribari C (2018). Organ injury scaling 2018 update: Spleen, liver, and kidney. J Trauma Acute Care Surg, United States.

[4] Coccolini F, Montori G, Catena F, Di Saverio S, Biffl W, Moore EE, et al. Liver trauma: WSES position paper. World J Emerg Surg; 2015;10: 39 10.1186/s13017-015-0030-9 PMC4548919 26309445

[5] Coccolini F, Catena F, Moore EE, Ivatury R, Biffl W, Peitzman A, et al. WSES classification and guidelines for liver trauma. World J Emerg Surg. England; 2016;11:50. 10.1186/s13017-016-0105-2 PMC5057434 27766112

[6] Coccolini F, Montori G, Catena F, Kluger Y, Biffl W, Moore EE, et al. Splenic trauma: WSES classification and guidelines for adult and pediatric patients. World J Emerg Surg. England; 2017;12:40. 10.1186/s13017-017-0151-4 PMC5562999 28828034

[7] Coccolini F, Stahel PF, Montori G, Biffl W, Horer TM, Catena F, et al. Pelvic trauma: WSES classification and guidelines. World J Emerg Surg. England; 2017;12:5. 10.1186/s13017-017-0117-6 PMC5241998 28115984

[8] Coccolini F, Catena F, Kluger Y, Sartelli M, Baiocchi G, Ansaloni L, et al. Abdominopelvic trauma: from anatomical to anatomo-physiological classification. World J Emerg Surg. England; 2018;13:50. 10.1186/s13017-018-0211-4 PMC6208045 30450123

[9] Coccolini F, Fugazzola P, Morganti L, Ceresoli M, Magnone S, Montori G, et al. The World Society of Emergency Surgery (WSES) spleen trauma classification: a useful tool in the management of splenic trauma. World J Emerg Surg. England; 2019;14:30. 10.1186/s13017-019-0246-1 PMC6580626 31236130

[10] Coccolini F, Kobayashi L, Kluger Y, Moore EE, Ansaloni L, Biffl W, et al. Duodeno-pancreatic and extrahepatic biliary tree trauma: WSES-AAST guidelines. World J Emerg Surg. England; 2019;14:56. 10.1186/s13017-019-0278-6 PMC6907251 31867050

[11] Coccolini F, Moore EE, Kluger Y, Biffl W, Leppaniemi A, Matsumura Y, et al. Kidney and uro-trauma: WSES-AAST guidelines. World J Emerg Surg. England; 2019;14:54. 10.1186/s13017-019-0274-x PMC6886230 31827593

[12] Pereira BMT, Chiara O, Ramponi F, Weber DG, Cimbanassi S, De Simone B, et al. WSES position paper on vascular emergency surgery. World J Emerg Surg. England; 2015;10:49. 10.1186/s13017-015-0037-2 PMC4618918 26500690

[13] Moore EE, Cogbill TH, Jurkovich GJ, Shackford SR, Malangoni MA, Champion HR (1995). Organ injury scaling: spleen and liver (1994 revision). J Trauma United States.

[14] Moore EE, Cogbill TH, Jurkovich GJ, McAninch JW, Champion HR, Gennarelli TA (1992). Organ injury scaling. III: Chest wall, abdominal vascular, ureter, bladder, and urethra. J Trauma. United States.

[15] Moore EE, Jurkovich GJ, Knudson MM, Cogbill TH, Malangoni MA, Champion HR (1995). Organ injury scaling. VI: Extrahepatic biliary, esophagus, stomach, vulva, vagina, uterus (nonpregnant), uterus (pregnant), fallopian tube, and ovary. J Trauma. United States.

[16] Moore EE, Cogbill TH, Malangoni MA, Jurkovich GJ, Champion HR, Gennarelli TA (1990). Organ injury scaling, II: Pancreas, duodenum, small bowel, colon, and rectum. J Trauma United States.

[17] Moore EE, Malangoni MA, Cogbill TH, Shackford SR, Champion HR, Jurkovich GJ (1994). Organ injury scaling. IV: Thoracic vascular, lung, cardiac, and diaphragm. J Trauma. United States.

[18] Coccolini F, Sartelli M, Kluger Y, Pikoulis E, Karamagioli E, Moore EE (2020). COVID-19 the showdown for mass casualty preparedness and management: the Cassandra Syndrome. World J Emerg Surg.

[19] Advanced Trauma Life Support ® (ATLS ®), 10th Edition, 2018, American College of Surgeons, The Committee on Trauma.

[20] Debus F, Lefering R, Frink M, Kühne CA, Mand C, Bücking B, et al. Numbers of Severely Injured Patients in Germany. A Retrospective Analysis From the DGU (German Society for Trauma Surgery) Trauma Registry. Dtsch Arztebl Int. 2015;112:823–9. 10.3238/arztebl.2015.0823 PMC4711294 26754119

[21] Lutz N, Vandermensbrugghe NG, Dolci M, Amiet V, Racine L, Carron P-N (2014). Pediatric emergencies admitted in the resuscitation room of a Swiss university hospital. Pediatr Emerg Care United States.

[22] Wesson DE (2012). Pediatric trauma centers: coming of age. Texas Hear Inst J.

[23] Osler TM, Vane DW, Tepas JJ, Rogers FB, Shackford SR, Badger GJ (2001). Do pediatric trauma centers have better survival rates than adult trauma centers? An examination of the National Pediatric Trauma Registry. J Trauma United States.

[24] Choi PM, Hong C, Woods S, Warner BW, Keller MS (2016). Early impact of American College of Surgeons-verification at a level-1 pediatric trauma center. J Pediatr Surg United States.

[25] Livingston DH, Lavery RF, Passannante MR, Skurnick JH, Baker S, Fabian TC (2001). Free fluid on abdominal computed tomography without solid organ injury after blunt abdominal injury does not mandate celiotomy. Am J Surg United States.

[26] Mehall JR, Ennis JS, Saltzman DA, Chandler JC, Grewal H, Wagner CW (2001). Prospective results of a standardized algorithm based on hemodynamic status for managing pediatric solid organ injury. J Am Coll Surg United States.

[27] Notrica DM, Eubanks JW, Tuggle DW, Maxson RT, Letton RW, Garcia NM (2015). Nonoperative management of blunt liver and spleen injury in children: Evaluation of the ATOMAC guideline using GRADE. J Trauma Acute Care Surg United States.

[28] St Peter SD, Sharp SW, Snyder CL, Sharp RJ, Andrews WS, Murphy JP (2011). Prospective validation of an abbreviated bedrest protocol in the management of blunt spleen and liver injury in children. J Pediatr Surg United States.

[29] Strohm PC, Zwingmann J, Bayer J, Neumann MV, Lefering R, Schmal H (2018). Differences in the outcome of seriously injured children depending on treatment level. Unfallchirurg Germany.

[30] Hall JR, Reyes HM, Meller JL, Loeff DS, Dembek R (1996). The outcome for children with blunt trauma is best at a pediatric trauma center. J Pediatr Surg United States.

[31] Potoka DA, Schall LC, Gardner MJ, Stafford PW, Peitzman AB, Ford HR (2000). Impact of pediatric trauma centers on mortality in a statewide system. J Trauma United States.

[32] Ingram M-CE, Siddharthan R V, Morris AD, Hill SJ, Travers CD, McKracken CE, et al. Hepatic and splenic blush on computed tomography in children following blunt abdominal trauma: Is intervention necessary? J Trauma Acute Care Surg. United States; 2016;81:266–70. 10.1097/TA.0000000000001114 27257698

[33] Stylianos S (2005). Outcomes from pediatric solid organ injury: role of standardized care guidelines. Curr Opin Pediatr United States.

[34] Reichert M, Sartelli M, Weigand MA, Doppstadt C, Hecker M, Reinisch-Liese A (2020). Impact of the SARS-CoV-2 pandemic on emergency surgery services-a multi-national survey among WSES members. World J Emerg Surg.

[35] Reichert M, Sartelli M, Weigand MA, Hecker M, Oppelt PU, Noll J (2022). Two years later: Is the SARS-CoV-2 pandemic still having an impact on emergency surgery? An international cross-sectional survey among WSES members. World J Emerg Surg.

[36] Eysenbach G. Improving the quality of Web surveys: the Checklist for Reporting Results of Internet E-Surveys (CHERRIES). J. Med. Internet Res. 2004. p. e34. 10.2196/jmir.6.3.e34 PMC1550605 15471760

[37] American College of Surgeons. Resources for Optimal Care of the Injured Patient, 2014 Standards. 2014. https://www.facs.org.

[38] American College of Surgeons. Resources for Optimal Care of the Injured Patient, 2022 Standards. 2022. https://www.facs.org.

[39] Coccolini F, Coimbra R, Ordonez C, Kluger Y, Vega F, Moore EE (2020). Liver trauma: WSES 2020 guidelines. World J Emerg Surg.

