# Supplementary material for: Pediatric trauma and emergency surgery: an international cross-sectional survey among WSES members
Source: World J Emerg Surg. 2023 Jan 13;18:6. doi: 10.1186/s13017-022-00473-5 (PMC9840264; doi:10.1186/s13017-022-00473-5)
Supplement: Supplementary file 1 — Additional file 1. The WSES pediatric emergency surgery collaboration group (only those who agree are listed as collaborators). [file 13017_2022_473_MOESM1_ESM.docx]

***The WSES pediatric emergency surgery collaboration group*** (only those who agree are listed as collaborators)

**Name** (alphabetic order), **Affiliation**

Agron Dogjani, University of Medicine of Tirana, Tirana, Albania

Akira Kuriyama, Emergency and Critical Care Center, Kurashiki Central Hospital, Japan

Alberto Porcu, Università degli Studi di Sassari, Italy

Aleix Martínez-Pérez, Department of General and Digestive Surgery, Hospital Universitario Doctor Peset, Valencia, Spain

Alessandro Coppola, General Surgery, Fondazione Policlinico Universitario Campus Bio-Medico, Rome, Italy

Alessandro Spolini, UOC Chirurgia Generale Sondrio ASST Valtellina-Alto Lario, Italy

Alessio Giordano, General Surgery unit, Nuovo Ospedale S. Stefano, Prato, Italy

Alexandros Kyriakidis, General Hospital of Amfissa, Greece

Ali Yasen Y Mohamedahmed, Sandwell and West Birmingham NHS trust, United Kingdome

Anastasia Vasilopoulou, Trauma and Orthopedic Department, Korgialenio-Benakio- Hellenic Red Cross Hospital, Athens, Greece

Andee Dzulkarnaen Zakaria, Department of Surgery, School of Medical Sciences & Hospital USM, Universiti Sains Malaysia, Malaysia

Andrea Balla, UOC of General and Minimally Invasive Surgery, Hospital “San Paolo”, Largo Donatori del Sangue 1, Civitavecchia, Rome, Italy

Andreas Fette, PS_SS, Weissach im Tal, Germany

Andrey Litvin, Department of Surgical Disciplines, Immanuel Kant Baltic Federal University, Regional Clinical Hospital, Kaliningrad, Russia

Anna Guariniello, Santa Maria delle Croci Hospital, Ravenna, Italy

Arda Isik, Istanbul medeniyet university, Turkey

Aristotelis Kechagias, Department of General Surgery, Kanta-Häme Central Hospital, Finland

Ashrarur Rahman Mitul, Bangladesh Shishu Hospital & Institute, Bangladesh

Belinda De Simone, Department of Emergency, digestive and metabolic minimally invasive surgery, Poissy and Saint Germain en Laye Hospitals, Ile de France, France

Biagio Zampogna, Department of Orthopaedic and Trauma Surgery, Campus Bio-Medico University of Rome, Rome, Italy

Bruno Sensi, Policlinico tor vergata, Rome, Italy

Carlo Gazia, Istituto Nazionale Tumori Regina Elena, Italy

Charalampos Seretis, General University Hospital of Patras, Greece

Cristine Brooke

Davide Luppi, General And Emergency Surgery, ASMN IRCCS Reggio Emilia, Italy

Diego Coletta, AO Ospedali Riuniti Marche Nord, Department of General Surgery, Italy

Diego Sasia, General and Oncological Surgery Unit, Santa Croce and Carle Hospital, Cuneo, Italy

Diletta Corallino, Policlinico Umberto I Rome, Italy

Dimitrios Chatzipetris, Department of Surgery, Metaxa Cancer Hospital, Piraeus, Greece

Dimitrios Schizas, First Department of Surgery, Laikon General Hospital, University of Athens, Athens, Greece

Eftychios Lostoridis, Kavala General Hospital, Kavala, Greece

Elmuiz A. Hsabo, Manchester University NHS Foundation Trust, United Kingdom

Emmanouil Kaouras, Department of Surgery, Metaxa Cancer Hospital, Piraeus, Greece

Emmanuel Schneck, Department of Anesthesiology, Operative Intensive Care Medicine and Pain Therapy, University Hospital of Giessen, Rudolf-Buchheim-Strasse 7, Giessen, Germany

Enrico Pinotti, Policlinico San Pietro, Italy

Evgeni Dimitrov, Department of Surgical Diseases, University Hospital "Prof. Dr. Stoyan Kirkovich" Stara Zagora, Bulgaria

Fabrizio D'Acapito, Chirurgia Generale e Terapie ocnologiche Avanzate, Morgagni-Pierantoni Hospital, Forlì, AUSL Romagna, Italy

Federica Saraceno, UOC of General and Minimally Invasive Surgery, Hospital „San Paolo“, Largo Donatori del Sangue 1, Civitavecchia, Rome, Italy

Fikri Abu-Zidan, Department of Surgery, College of Medicine, UAE University, Al-Ain, United Arab Emirates

Francesca Maria Silvestri

Francesco Favi, U.O.C. Chirurgia Gerenale e d’Urgenza, Dipartimento Chirurgico e Grandi Traumi - Ospedale “M. Bufalini” Cesena AUSL della Romagna, Italy

Francesco Fleres, General Surgery Unit - ASST Valtellina e Alto Lario, Sondrio Hospital, Sondrio, Italy

Francesk Mulita, Department of Surgery, General University Hospital of Patras, Greece

Gabriela Nita, Sant'Anna Hospital, AUSL Reggio Emilia, Italy

Gennaro Martines, Azienda Ospedaliero Universitaria Policlinico Bari, Italy

Gennaro Mazzarella, Department of Emergency Surgery, Sapienza University of Rome, Rome, Italy

Gennaro Perrone, Department of emergency surgery, Parma Maggiore Hospital, Parma, Italy

Giorgio Giraudo, Department of Surgery ASO Santa Croce e Carle Cuneo, Italy

Giulia Bacchiocchi, Tor Vergata University, Rome, Italy

Giulio Argenio, UOC Chirurgia d'Urgenza, AOU San Giovanni di Dio e Ruggi d'Aragona, Salerno, Italy

Giuseppe Brisinda, Department of Surgery, Fondazione Policlinico Universitario A Gemelli, IRCCS and Università Cattolica S. Cuore, Rome, Italy

Giuseppe Currò, AOU Mater Domini Catanzaro - Magna Graecia University of Catanzaro, Italy

Giuseppe Palomba, Università Federico II di Napoli, Italy

Gustavo P. Fraga, Division of Trauma Surgery, School of Medical Sciences, University of Campinas (Unicamp), Brazil

Hytham K. S. Hamid, Kuwaiti Specialized Hospital, Khartoum, Sudan

Ioannis Katsaros, Department of Surgery, Metaxa Cancer Hospital, Pireaus, Greece

Ionut Negoi, General Surgery Department, Carol Davila University of Medicine and Pharmacy Bucharest, Emergency Hospital of Bucharest, Romania

Joel Noutakdie Tochie, Department of Emergency Medicine, Anaesthesiology, Critical Care Medicine, Laquintinie Hospital of Douala, Douala, Cameroon

Justin Davies, Addenbrooke’s Hospital, Cambridge University Hospitals NHS Foundation Trust, United Kingdom

Kenneth Y. Y. Kok, Pengiran Anak Puteri Rashidah Sa´adatul Bolkiah Institue of Health Sciences, Universiti Brunei darussalam, Brunei

Konstantinos G. Apostolou, Department of General and Endocrine Surgery, Athens Medical Center, Palaio Faliro, Athens, Greece

Konstantinos Lasithiotakis, Department of Surgery, University Hospital of Heraklion, Crete, Greece

Konstantinos Tsekouras, Sismanogleio General Hospital, Athens, Greece

Larysa Sydorchuk, Bukovinian State Medical University, Ukraine

Leandro Siragusa, Department of Surgery, Università degli studi di Roma „Tor Vergata“, Italy

Leonardo Solaini, Department of Medical and Surgical Sciences, University of Bologna, Morgagni Pierantoni Hospital, Forlì, Italy

Luca Ferrario, General Surgery Trauma Team ASST-GOM Niguarda, Milan, Italy

Luis Buonomo, Hospital Zonal de Agudos "Dr. Alberto Balestrini", Argentina

Maciej Walędziak, Military Institute of Medicine, Department of General Surgery, Poland

Mahir Gachabayov, Vladimir City Emergency Hospital, Russia

Maloni Bulanauca, Department of Surgery, Labasa Hospital, Fiji

Manish Kumar Agrawal, King George’s Medical University Lucknow, India

Marco Ceresoli, University of Milano-Bicocca, Italy

Maria Chiara Ranucci, Azienda ospedaliera Santa Maria di Terni, Italy

Maria Petridou, Department of Pediatric Surgery, Ippokration General Hospital of Thessaloniki, Thessaloniki, Greece

Mario D'Oria, Division of Vascular and Endovascular Surgery, Cardiovascular Department, University Hospital of Trieste, Italy

Massimiliano Veroux, General Surgery, University Hospital of Catania, Department of Medical and Surgical Sciences and Advanced Technologies, University of Catania, Italy

Maximos Frountzas, First Propaedeutic Department of Surgery, National and Kapodistrian University of Athens, Medical School, Hippocration General Hospital of Athens, Greece

Michel Paul Johan Teuben, Cantonal Hospital Frauenfeld, Switzerland

Miklosh Bala, Department of General Surgery, Hadassah Medical Center and Faculty of Medicine, Hebrew University of Jerusalem, Israel

Mirja Amadea Minger, Department of Pediatric Surgery, Inselspital, Bern University Hospital, University of Bern, Bern, Switzerland

Miroslava Gonçalves, Centro Hospitalar Universitário Lisboa Norte - Hospital de Santa Maria, Lisboa, Portugal

Natasha Sharma

Nicolò Tamini, ASST Monza - Ospedale San Gerardo, Italy

Noushif Medappil, Aster Malabar Institute of Medical Sciences (Aster MIMS - Calicut), India

Orestis Ioannidis, 4th Department of Surgery, Medical School, Aristotle University of Thessaloniki, General Hospital “George Papanikolaou”, Thessaloniki, Greece

Pietro Bisagni, Department of Surgery, ASST di Lodi, Lodi, Italy

Razrim Rahim, Universiti Sains Islam Malaysia, Malaysia

Ricardo Alessandro Teixeira Gonsaga, Centro Universitário Padre Albino, Catanduva (SP), Brazil

Roberta Ragozzino, General surgery and Trauma Team, ASST Niguarda, Milano, Piazza Ospedale Maggiore 3, Milan, Italy

Roberto Bini, ASST Niguarda Hospital, Italy

Roberto Cammarata, Campus Biomedico University of Rome, Italy

Ruslan Sydorchuk, General Surgery Department, Regional Acute and Emergency Hospital Chernivtsi, Ukraine

Salomone Di Saverio, Ospedale Madonna del Soccorso San benedetto del Tronto, Italy

Selmy S. Awad, Mansoura university hospitals, Mansoura University, Egypt

Semra Demirli Atici, University of Health Sciences Tepecik Training and Research Hospital, Turkey

Serhat Meric, General surgery department, Bağcılar training and research hospital, Istanbul, Turkey

Sharfuddin Chowdhury, King Saud Medical City, Riyadh, Saudi Arabia

Sofia Xenaki, Department of General Surgery, University Hospital of Heraklion Crete, Greece

Tadeja Pintar, Surgery Department, UMC Ljubljana, Ljubljana, Sloviena

Teresa Perra, Università degli Studi di Sassari, Italy

Timothy C. Hardcastle, IALCH Trauma and UKZN Department of Surgery, Durban and University of KwaZulu-Natal, South Africa

Valerio Voglino, Ospedale Pediatrico Bambino Gesù, Roma, Italy

Varut Lohsiriwat, Department of Surgery, Faculty of Medicine Siriraj Hospital, Mahidol University, Bangkok, Thailand

Victor Kong, Department of Surgery, University of KwaZulu Natal, Durban, South Africa

Voskidis Christos, Pediatric Surgery, Greece

Wietse Zuidema, Amsterdam University Medical Center, Amsterdam, Netherlands
